# 

**Published:** 1881-08

**Authors:** 


*The Fifth Annual Session of the Missouri Institute of Homœopathy*, will be held at Sweet Springs, Mo., August 3d and 4th, 1881. Members and their families will be entertained at the Sweet Springs Hotel, at $2 per day. These Springs are growing into great favor, and a large and very interesting meeting is expected.

WM. D. FOSTER, M. D., *Secretary.*

---

a12

VOL. I. AUGUST, 1881. No. 8.

THE

### HOMŒOPATHIC PHYSICIAN,

A MONTHLY JOURNAL OF MEDICAL SCIENCE.

*"Magna est veritas, et prævalebit."*

0

E. J. LEE, M. D.

EDITOR.

CONTENTS:

(See inside of Cover.)

CONTRIBUTORS:

Drs. H. C. Allen, T. F. Allen, Edward Bayard, J. B. Bell, E. W. Berridge, A. C. Cowperthwaite, Ad. Fellger, W. A. Hawley, John Hall, W. H. Jenny, Ad. Lippe, C. Lippe, Malcolm MacFarlan, Thos. Moore, C. F. Nichols, C. Pearson, T. F. Pomeroy, C. Carleton Smith, Thos. Skinner, P. P. Wells, Wm. P. Wesselhoeft, David Wilson, (London), T. P. Wilson, and many others.

NOTICE.

***All articles for publication, books for review, business communications, etc., must be sent to Editor, 2109 Chestnut Street, Philadelphia.***

N. B.—Authors of accepted Papers are furnished with copies of the number in which their articles appear; application for such copies to be made when sending papers. Any irregularity in the receipt of this Journal should be promptly reported to Editor.

A prize of fifty dollars will be awarded to Author of the best Essay printed in this Journal for the year 1881. To be adjudged by three physicians of note.

